# Modified Photochemical Reflectance Indices as New Tool for Revealing Influence of Drought and Heat on Pea and Wheat Plants

**DOI:** 10.3390/plants11101308

**Published:** 2022-05-14

**Authors:** Ekaterina Sukhova, Lyubov Yudina, Anastasiia Kior, Dmitry Kior, Alyona Popova, Yuriy Zolin, Ekaterina Gromova, Vladimir Sukhov

**Affiliations:** Department of Biophysics, N.I. Lobachevsky State University of Nizhny Novgorod, 603950 Nizhny Novgorod, Russia; n.catherine@inbox.ru (E.S.); lyubovsurova@mail.ru (L.Y.); nastay2903@bk.ru (A.K.); dimakior@mail.ru (D.K.); silverkumiho@mail.ru (A.P.); uchebnayap.zolin@gmail.com (Y.Z.); kater333@inbox.ru (E.G.)

**Keywords:** modified photochemical reflectance indices, PRI, water shortage, soil drought, short-term heat, photosynthetic changes, pea, wheat

## Abstract

In environmental conditions, plants can be affected by the action of numerous abiotic stressors. These stressors can induce both damage of physiological processes and adaptive changes including signaling-based changes. Development of optical methods of revealing influence of stressors on plants is an important task for plant investigations. The photochemical reflectance index (PRI) based on plant reflectance at 531 nm (measuring wavelength) and 570 nm (reference wavelength) can be effective tool of revealing plant stress changes (mainly, photosynthetic changes); however, its efficiency is strongly varied at different conditions. Earlier, we proposed series of modified PRIs with moderate shifts of the measuring wavelength and showed that these indices can be effective for revealing photosynthetic changes under fluctuations in light intensity. The current work was devoted to the analysis of sensitivity of these modified PRIs to action of drought and short-term heat stress. Investigation of spatially-fixed leaves of pea plants showed that the modified PRI with the shorter measuring wavelength (515 nm) was increased under response of drought and heat; by contrast, the modified PRI with the longer wavelength (555 nm) was decreased under response to these stressors. Changes of investigated indices could be related to parameters of photosynthetic light reactions; however, these relations were stronger for the modified PRI with the 555 nm measuring wavelength. Investigation of canopy of pea (vegetation room) and wheat (vegetation room and open-ground) supported these results. Thus, moderate changes in the measuring wavelengths of PRI can strongly modify the efficiency of their use for the estimation of plant physiological changes (mainly photosynthetic changes) under action of stressors. It is probable that the modified PRI with the 555 nm measuring wavelength (or similar indices) can be an effective tool for revealing photosynthetic changes induced by stressors.

## 1. Introduction

In environmental conditions, plants can be affected by actions of numerous abiotic stressors including drought [1,2], heat [3,4], low temperature [5], excess precipitation [6], high intensity of light [7], etc.; it should be noted that soil drought and heat (which often accompanies this drought) are crucial factors limiting crops of plants at their cultivation [1,8,9,10]. Abiotic stressors can induce extensive physiological changes; it is known that photosynthesis, which is a basis of productivity, is an important target of action of these stressors including drought [11,12,13], heat [14,15], or fluctuations in light intensity [16,17].

It is known that the influence of drought on photosynthesis is mainly caused by a decrease of CO_2_ flux into the stroma of chloroplasts of mesophyll cells, which can be mediated by changes in concentration of abscisic acid (ABA) [18,19,20]. The influence of ABA on the CO_2_ flux is based on closing of stomata [18,20,21,22,23] and a decrease of CO_2_ conductance of mesophyll [24,25] caused by inactivation of H^+^-ATP-ase in the plasma membrane. Suppression of photosynthetic dark reactions caused by the decrease of CO_2_ flux into the stroma of chloroplasts can be a reason of changes in parameters of photosynthetic light reactions [25,26]; particularly, stimulation of the non-photochemical quenching of chlorophyll fluorescence (NPQ) and cyclic electron flow around photosystem I can be observed. It is interesting that these processes (increasing NPQ and cyclic flow) can be considered as protective mechanisms for photosynthetic machinery [16,27,28,29]. However, the suppression of photosynthetic dark reactions and further over-reduction of the electron transport chain in chloroplasts can also stimulate production of reactive oxygen species (ROS) [30,31], which are considered to be both signal molecules and agents damaging photosynthetic machinery and other cell structures.

Heat stress, which can be related to the development of the soil drought, also damages photosynthetic machinery (or suppresses reparation of this machinery) [14,15,32] and induces stress signals participating in the protection of plants [33]. Particularly, it is known [34] that increased temperatures can stimulate production of H_2_O_2_ by Rubisco; this response can participate in both the photosynthetic damage and ROS signaling [31]. Additionally, heat stress can increase tolerance of photosynthetic machinery to increased temperatures through the generation of local electrical responses [33].

Thus, drought and heat-induced photosynthetic responses in plants can be the result of intricate interactions between photosynthetic damages, processes of signaling, and adaptive changes. Considering this point, early revealing the photosynthetic responses can be important for protection of plants under actions of stressors and prediction of plant productivity. Measurements of plant reflectance at different spectral bands and calculation of reflectance indices (RIs) are important methods of this revealing [35]. Particularly, there are RIs (e.g., the normalized difference vegetation index (NDVI), normalized pigment chlorophyll ratio index (NPCI), MERIS terrestrial chlorophyll index (MTCI) and others [36,37]) that can be strongly correlated with photosynthetic parameters in plants.

A photochemical reflectance index (PRI(531,570)) developed by Gamon et al. [38] can be sensitive to very fast photosynthetic changes (minutes, seconds, and, even, hundreds of millisecond) [38,39,40,41,42,43,44]. Equation (1) can be used for PRI(531,570) calculation [42,45,46,47]:

(1) $$\mathrm{PRI}(531,570)=\frac{R_{531}-R_{570}}{R_{531}+R_{570}},$$

where R_531_ and R_570_ are the reflectance at 531 nm (the measuring wavelength) and 570 nm (the reference wavelength), respectively. It is traditionally considered [35,38,40,45,46,47,48] that fast changes in R_531_ are mainly related to transitions in the xanthophyll cycle; however, induction of the light scattering in the green–yellow spectral range, which is probably related to chloroplast shrinkage [41], can also participate in development of these PRI(531,570) changes [35,40,49,50]. Slow changes in PRI(531,570) are caused by changes in ratio between concentrations of carotenoids and chlorophylls and changes in the total pool size of xanthophylls [51,52,53].

The dependence value of PRI(531,570) on the de-epoxidation of violaxanthin to zeaxanthin [35,38,40,45,46,47] is the basis of relations between PRI(531,570) and photosynthetic parameters because the xanthophyll cycle is an important mechanism of NPQ [54,55] depended on acidification of the lumen of chloroplasts. The chloroplast shrinkage [41,42] (and the light scattering in the green–yellow spectral range [56,57,58]) is also stimulated by this acidification and can support the relation between the photochemical reflectance index and photosynthetic parameters. There are works showing strong relations between PRI(531,570) and these parameters under different intensities of light, development of drought, action of heat, propagations of long-distance electrical signals, and under action of other factors [38,39,41,44,59,60].

However, meta-analyses of literature data showed high variability of relations between the photochemical reflectance index and photosynthetic parameters [42,46,47]. It is important that directions of these relations can also be changed; for example, negative correlations between PRI(531,570) and the energy-dependent component of NPQ are observed under high intensity of light, but positive correlations between these parameters can be observed under a soil water shortage [43,44]. These effects make difficult using PRI(531,570) for revealing photosynthetic stress changes in plants and require the development of new methods of application of the photochemical reflectance index for revealing stress changes in photosynthetic processes.

Earlier, we showed that using light-induced changes in PRI(531,570) can increase correlations between the photochemical reflectance index and photosynthetic parameters [43,44,59]; works by other authors also support this [61,62,63]. However, measurements of the light-induced changes in PRI(531,570) requires the controlled artificial illumination [43,44] or at least two measurements of the photochemical reflectance index under natural illumination (before dawn and under sunlight) [64]. Both methods can limit the efficiency of using these light-induced changes in PRI(531,570) analysis of photosynthetic parameters under response of stressors.

Alternatively, we previously showed [50] that changes in R_531_ are related to two different components in changes in reflectance (the fast-relaxing and slow-relaxing changes in the photochemical reflectance index were revealed); this result is in accordance with Gamon et al. [40]. It is probable that the fast-relaxing changes are related to the chloroplast shrinkage and light scattering, and the slow-relaxing changes are related to transitions in the xanthophyll cycle [40]. Using modified photochemical reflectance indices (PRI(λ,570), where λ is the modified measuring wavelength in Equation (1) equaling 515, 525, 535, 545, or 555 nm), we revealed different relations between different PRI(λ,570) and photosynthetic parameters under increased intensities of light.

It is known that drought decreases CO_2_ influx through the closing of stomata [20,21] and a decrease of mesophyll conductance [24,25] and, thereby, suppresses the Calvin–Benson cycle; heat can directly inactivate Rubisco [34]. These processes should induce imbalance between a proton transport from the stroma to the lumen through the photosynthetic electron transport chain and a reverse transport of H^+^ through the H^+^-ATP-synthase because the reverse transport is suppressed [29]; the acidification of the lumen of chloroplasts is the result of this imbalance. Both fast-relaxing and slow-relaxing changes in the typical and modified photochemical reflectance indices should be dependent on the acidification [50] through the chloroplast shrinkage and transitions in the xanthophyll cycle, respectively. This means that modified PRI(λ,570) with λ equaling 515, 525, 535, 545, or 555 nm should be sensitive to drought- and heat-induced photosynthetic changes; however, different specific indices can be strongly or weakly affected by shifts in concentrations of carotenoids and chlorophylls. The last point is very important because these shifts induce slow changes in the typical photochemical reflectance index [51,52,53], which are the potential reason of the increase of this index under response to the stressors. The increase, which was observed in some investigations (e.g., the PRI(531,570) increase in pea plants under water shortage [44,59]), seems to be paradoxical because the decrease of the photochemical reflectance index is a typical plant response in action of stressors [47]. It is probable that elimination of this mechanism through using modified PRI(λ,570), which is weakly affected by shifts in concentration of pigments, can increase efficiency of using photochemical reflectance indices for revealing photosynthetic stress changes because the quantity of factors influencing the measured index can be minimized.

Thus, we hypothesized that using the modified photochemical reflectance indices [50] can be more effective for revealing photosynthetic changes under action of stressors (drought and heat) than using typical PRI(531,570); to evaluate this hypothesis was the main objective of the current work. For the solution of this task, we analyzed our unpublished results of experiments described in works [59] and [65], which were devoted to the analysis of reflectance indices in spatially-fixed leaves under short-term water shortage and heat stress and the same analysis in plant canopies under the prolonged soil drought. Wheat and pea were used as suitable model plants because the reflectance of these objects was investigated in our previous works [43,44,49,50,59,60,65]; the investigations simplified the current analysis. Additionally, both pea and wheat are important agricultural crops that can be strongly affected by drought and heat [1,2,3].

## 2. Results

### 2.1. Influence of Short-Term Water Shortage on Modified Photochemical Reflectance Indices in Spatially-Fixed Leaves of Pea Plants

The short-term water shortage was induced by termination of irrigation of the sand growth substrate because this termination induced a fast decrease of the water content in pea plants and suppression of photosynthetic activity.

**Figure 1:**
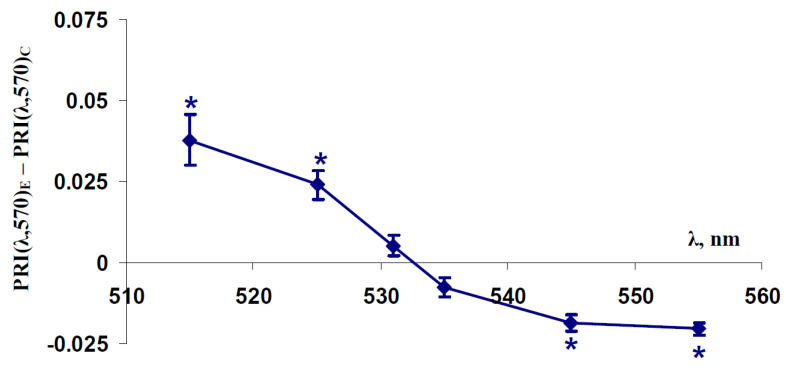
Dependence of difference between modified photochemical reflectance indices in leaves of pea plants under water shortage (PRI(λ,570)_E_) and these indices in leaves of control plants (PRI(λ,570)_C_) on wavelengths of the measuring reflected light (λ) (*n* = 5–6). PRI(λ,570)_E_ and PRI(λ,570)_C_ were measured after five days of water shortage. *, difference was significant (*p* < 0.05).

First, we analyzed changes in PRI(515,570), PRI(525,570), PRI(531,570), PRI(535,570), PRI(545,570), and PRI(555,570) induced by five days’ water shortage in spatially-fixed leaves of pea plants (Figure 1). Differences between modified photochemical reflectance indices in experimental (without irrigation) and control (with irrigation) pea plants were investigated. It was shown that PRI(λ,570) with λ equaling 515 and 525 nm were significantly increased under the water shortage; by contrast, PRI(λ,570) with λ equaling 535, 545 and 555 nm were significantly decreased. Significant changes in PRI(531,570) were absent.

This result shows that reflectance indices based on the slow-relaxing component of reflectance changes in the green spectral region (this component was mainly at 515 and 525 nm [50]) were increased by the water shortage, and the indices based on the fast-relaxing component of these changes (this component was mainly at 535, 545, and 525 nm [50]) were decreased under water-deficient conditions.

**Figure 2:**
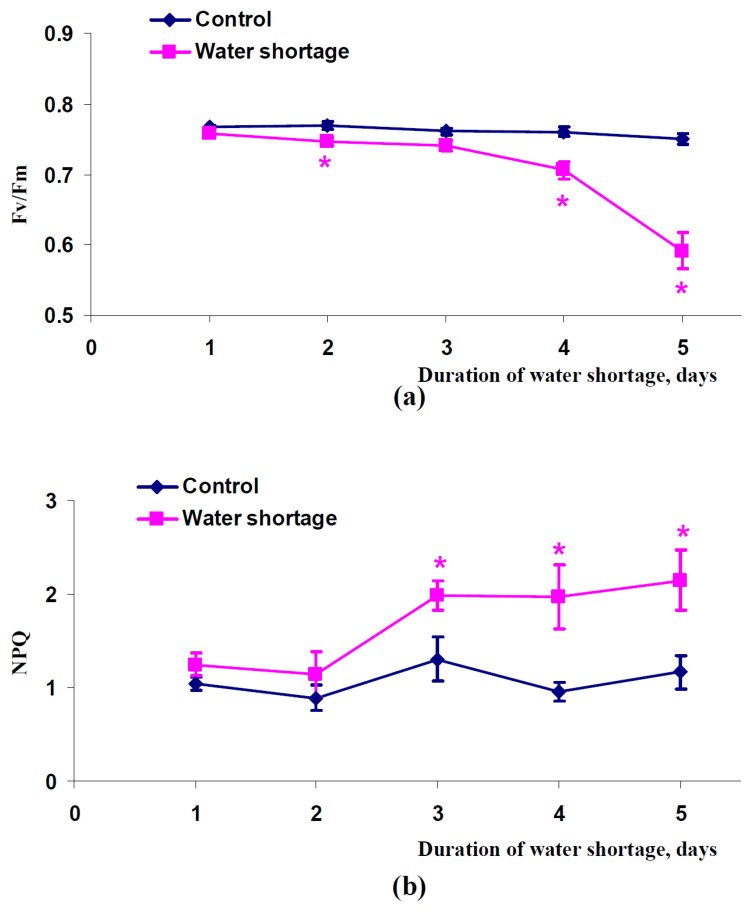
Dependence of the maximal quantum yield of photosystem II (Fv/Fm) (**a**) and non-photochemical quenching of chlorophyll fluorescence (NPQ) (**b**) in spatially fixed leaves of pea plants on duration of water shortage (*n* = 5–6). *, difference between experimental and control parameters was significant (*p* < 0.05).

It was supposed that changes in PRI(λ,570) were caused by water shortage-induced responses of photosynthetic processes. Considering this point, we analyzed dynamics of changes in the maximal quantum yield of photosystem II (Fv/Fm) (Figure 2a) and non-photochemical quenching in chlorophyll fluorescence (NPQ) (Figure 2b) during the short-term water shortage. It was shown that the water shortage induced a significant decrease of Fv/Fm, which had large magnitudes after four and five days without irrigation. Considering relations between Fv/Fm and photosynthetic machinery [66,67], the decrease of the maximal quantum yield of photosystem II showed damage to this machinery under water-deficit conditions. A significant increase of NPQ was observed after three, four, and five days of water shortage (Figure 2b). Considering the relation of NPQ to both the photodamage and mechanisms of protection of the photosynthetic machinery against action of stressors [16,28,55,68], it was probable that this result also showed induction of protective processes in photosynthetic machinery in plants under conditions of water deficit.

PRI(515,570) and PRI(555,570) had maximal magnitudes of the increase and decrease, respectively. As a result, these modified photochemical reflectance indices were used for analysis of dynamics of their changes in pea plants during development of the soil water shortage. Relations of PRI(515,570) and PRI(555,570) to photosynthetic parameters were also analyzed.

**Figure 3:**
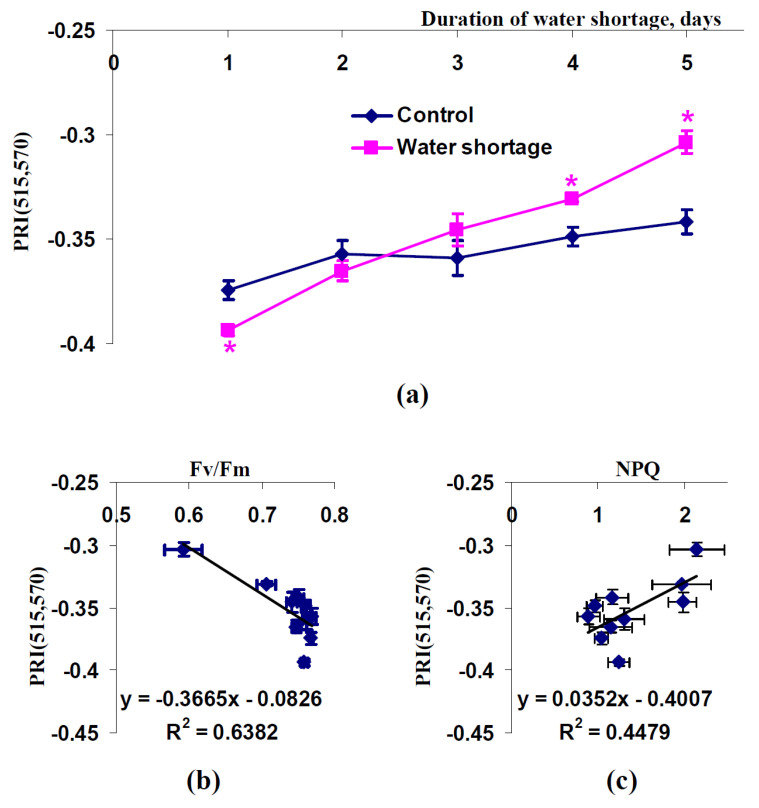
Dependence of the modified photochemical reflectance index calculated on the basis of 515 and 570 nm wavelengths (PRI(515,570)) in spatially-fixed leaves of pea plants on duration of water shortage (*n* = 5–6) (**a**) and dependences of average values of PRI(515,570) on Fv/Fm (**b**) and NPQ (**c**) (*n* = 10). Average values of Fv/Fm and NPQ from Figure 2 were used. R^2^ is the determination coefficient. *, difference between experimental and control indices was significant (*p* < 0.05).

It was shown (Figure 3a) that the water shortage stimulated the significant increase of PRI(515,570) after four and five days without irrigation. Analysis of dependence of average PRI(515,570) on average Fv/Fm during the water shortage (Figure 3b) showed a moderate linear relation between these parameters (R^2^ = 0.64). A scatter plot between average PRI(515,570) and average NPQ (Figure 3c) shows that the linear relation between these parameters was weak (R^2^ = 0.45).

**Figure 4:**
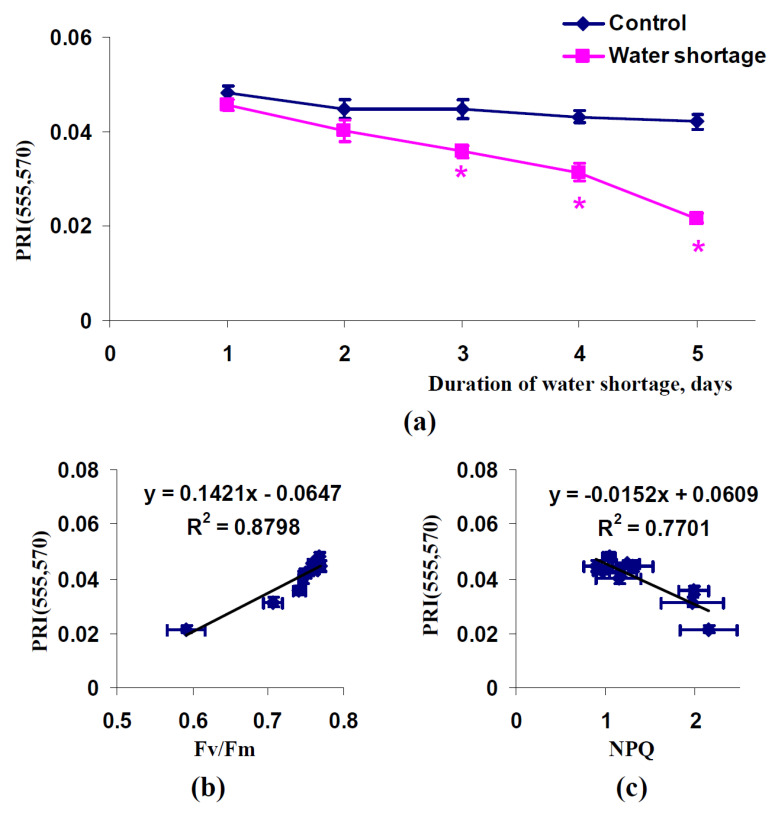
Dependence of the modified photochemical reflectance index calculated on the basis of 555 and 570 nm wavelengths (PRI(555,570)) in spatially-fixed leaves of pea plants on duration of water shortage (*n* = 5–6) (**a**) and dependences of average values of PRI(555,570) on Fv/Fm (**b**) and NPQ (**c**) (*n* = 10). Average values of Fv/Fm and NPQ from Figure 2 were used. R^2^ is the determination coefficient. *, difference between experimental and control indices was significant (*p* < 0.05).

Analysis of PRI(555,570) showed that this index was significantly decreased after three, four, and five days of soil water shortage (Figure 4a). It was noted that PRI(555,570) was relatively stable in control (irrigated) pea plants; i.e., this index could be considered as more effective for estimation of drought influence on plants.

Efficiency of PRI(555,570) was also supported by an analysis of scatter plots between this index and photosynthetic parameters. There were strong linear relations between PRI(555,570) and the maximal quantum yield of photosystem II (R^2^ = 0.88) (Figure 4b) and the non-photochemical quenching of chlorophyll fluorescence (R^2^ = 0.77) (Figure 4c). It is interesting that the linear relation between PRI(555,570) and Fv/Fm was stronger than the similar relation between PRI(555,570) and NPQ.

### 2.2. Influence of Short-Term Heat Stress on Modified Photochemical Reflectance Indices in Spatially-Fixed Leaves of Pea Plants

**Figure 5:**
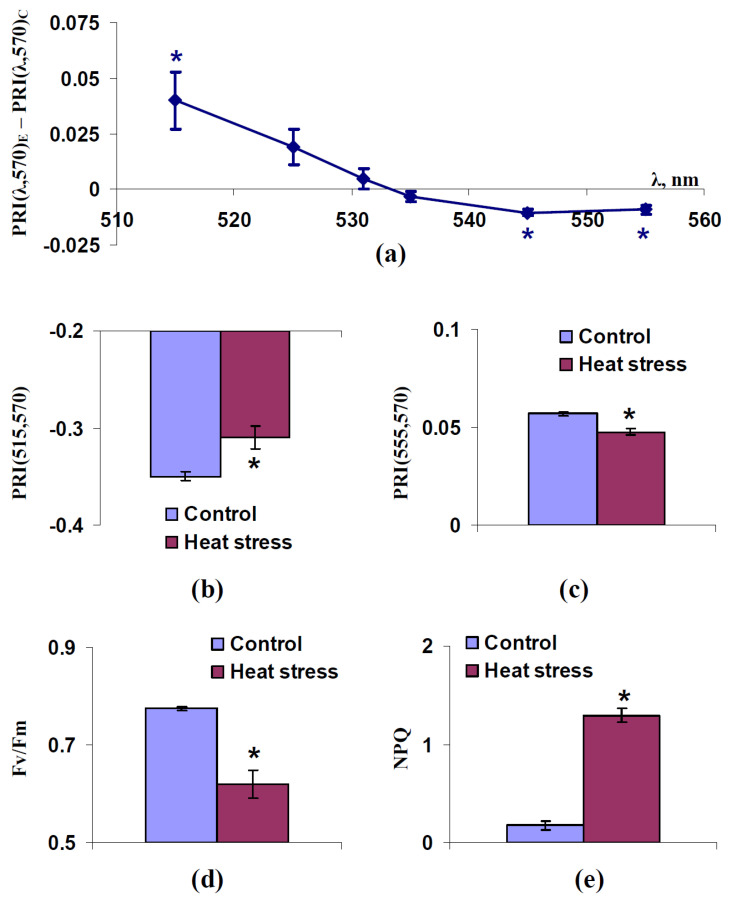
Dependence of difference between modified photochemical reflectance indices in spatially-fixed leaves of pea plants 1 h after termination of the short-term heat (46.5 °C, 30 min) (PRI(λ,570)_E_) and leaves of control plants (PRI(λ,570)_C_) on wavelengths of the measuring reflected light (λ) (**a**), and influence of the heat stress on PRI(515,570) (**b**), PRI(555,570) (**c**), Fv/Fm (**d**), and NPQ (**e**) (*n* = 5). *, difference was significant (*p* < 0.05).

In the next stage of the analysis, we investigated the influence of short-term heat stress on modified photochemical reflectance indices to compare this influence with the influence of water shortage on PRI(λ,570). It was shown (Figure 5a) that the difference between PRI(λ,570) in leaves of experimental (1 h after heat) and control (without heat stress) plants was positive at λ equaling 515 nm and negative at λ equaling 545 and 555 nm. Non-significant positive differences were also observed at 525 and 531 nm; a non-significant negative difference was observed at 535 nm.

Average values of PRI(515,570) and PRI(555,570) in control and experimental plants are shown in Figure 5b,c, respectively. It was noted that the increase of PRI(515,570) after action of heat corresponded to the decrease of Fv/Fm (Figure 5d) and the increase of NPQ (Figure 5e). The decrease of PRI(555,570) also corresponded to the Fv/Fm decrease and NPQ increase. This result was similar to the relations of PRI(515,570) and PRI(555,570) to photosynthetic parameters at water shortage.

### 2.3. Influence of Soil Drought on Modified Photochemical Reflectance Indices in Canopy of Pea and Wheat Plants

Analysis of influence of the soil drought on PRI(λ,570) measured in the canopy of plants included two variants of experiments: (i) wheat and pea plants were cultivated under conditions of a vegetation room and (ii) wheat plants were cultivated under open-ground conditions [65].

**Figure 6:**
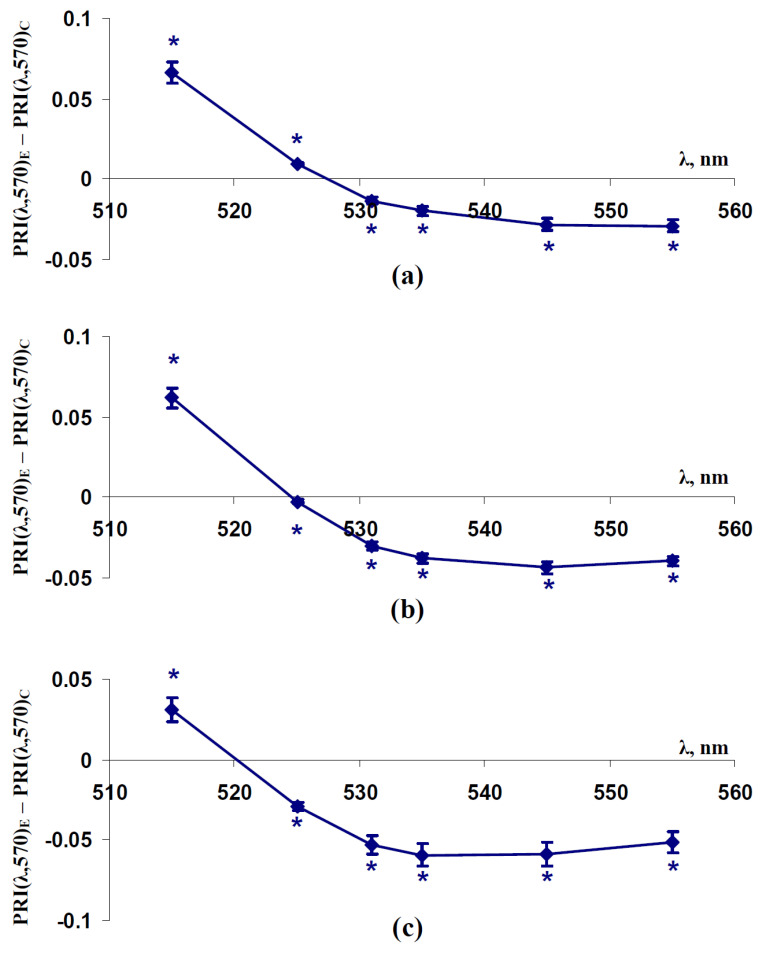
Dependences of difference between modified photochemical reflectance indices in canopy of plants under soil drought conditions (PRI(λ,570)_E_) and the canopy of control plants (PRI(λ,570)_C_) on wavelengths of the measuring reflected light (λ) in pea (**a**), wheat cultivated under conditions of a vegetation room (**b**), and wheat cultivated under open-ground conditions (**c**) (*n* = 45). PRI(λ,570)_E_ and PRI(λ,570)_C_ were measured after 13 (for pea plants cultivated under conditions of a vegetation room), 17 (for wheat plants cultivated under conditions of a vegetation room), and 11 days (for wheat plants cultivated under open-ground conditions) of this drought. *, difference was significant (*p* < 0.05).

Investigation of differences between PRI(λ,570) in control and experimental plants showed (Figure 6) that PRI(515,570) was significantly increased in all experimental variants at the final day of the soil drought. By contrast, PRI(531,570), PRI(535,570), PRI(545,570), and PRI(555,570) were decreased in these experimental variants. Changes in PRI(525,570) were positive in pea plants and negative in wheat plants. This result was qualitatively similar to results of analysis of pea plants with spatially-fixed leaves; however, PRI(531,570) (the typical photochemical reflectance index) was significantly decreased in all investigations of pea and wheat canopy. The last result was in good accordance with a decrease of PRI(531,570) under the action of stressors that was shown in many works (see, e.g., reviews [42,46,47]); however, this index was not significantly changed in experiments with spatially-fixed leaves (Figure 1).

**Figure 7:**
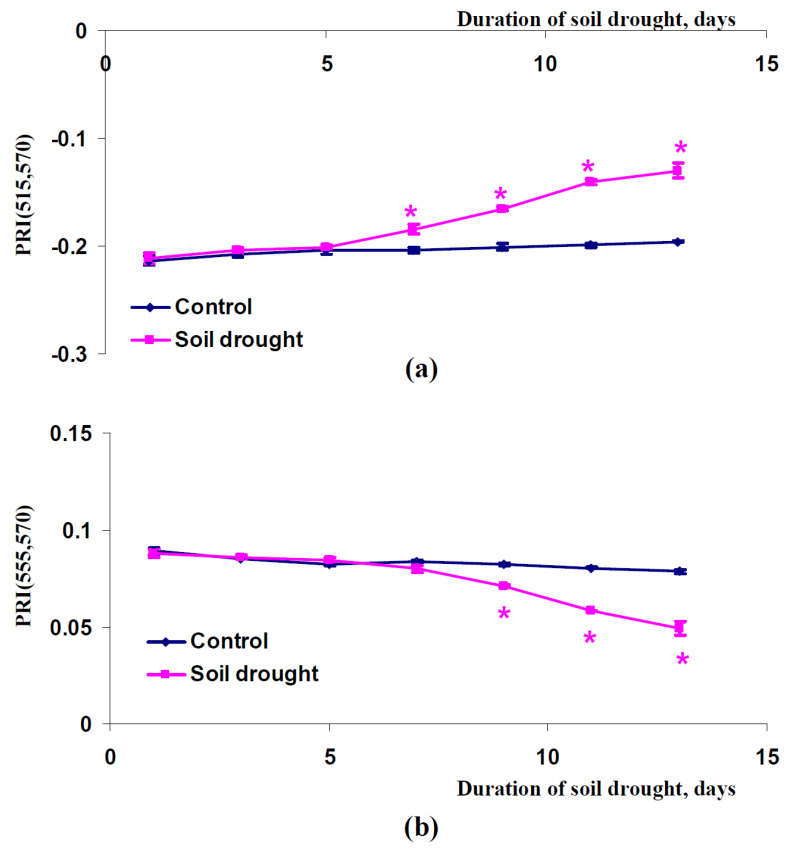
Dependences of the PRI(515,570) (**a**) and PRI(555,570) indices (**b**) in the canopy of pea plants cultivated under conditions of a vegetation room on the duration of the soil drought (*n* = 45). *, difference between experimental and control indices was significant (*p* < 0.05).

**Figure 8:**
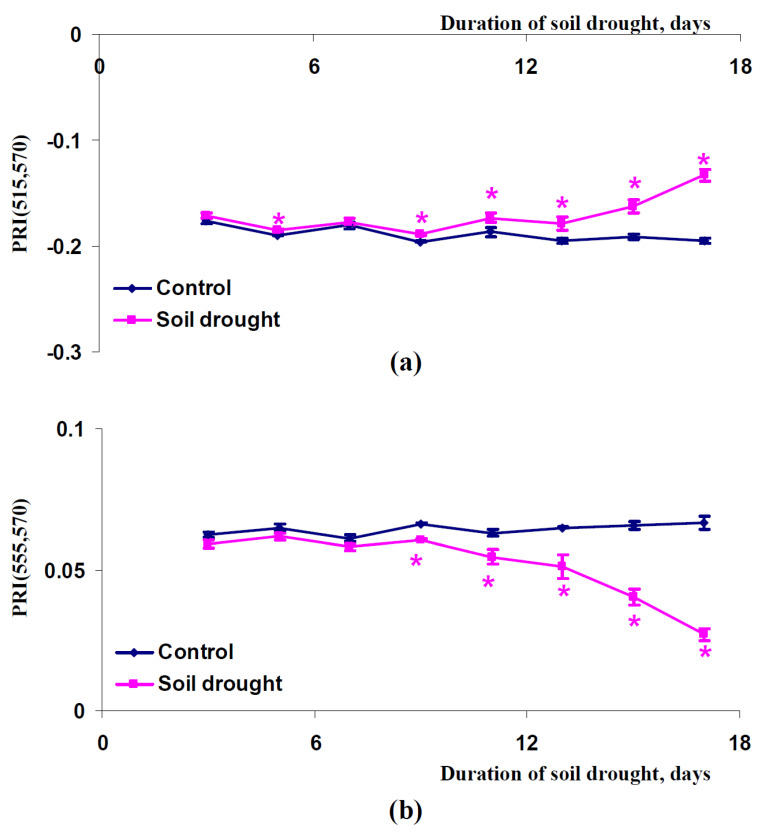
Dependences of the PRI(515,570) (**a**) and PRI(555,570) indices (**b**) in the canopy of wheat plants cultivated under conditions of a vegetation room on the duration of the soil drought (*n* = 45). *, difference between experimental and control indices was significant (*p* < 0.05).

**Figure 9:**
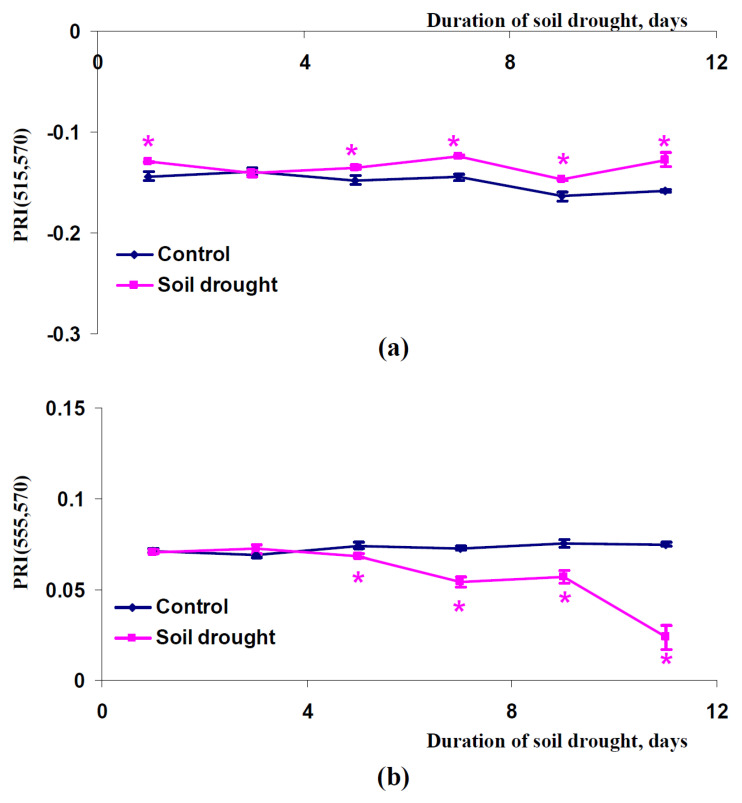
Dependences of the PRI(515,570) (**a**) and PRI(555,570) indices (**b**) in the canopy of wheat plants cultivated under open-ground conditions on the duration of the soil drought (*n* = 45). *, difference between experimental and control indices was significant (*p* < 0.05).

After that, we investigated dynamics of changes in PRI(515,570) and PRI(555,570) in canopies of pea plants, which were cultivated under conditions of a vegetation room (Figure 7), canopies of wheat plants, which were cultivated under conditions of a vegetation room (Figure 8), and canopies of wheat plants, which were cultivated under open-ground conditions (Figure 9). Similar results were shown in all experimental variants: PRI(515,570) was increased with increasing duration of the soil drought and PRI(555,570) decreased with increasing duration of this drought. The PRI(515,570) increases were initiated after seven days (pea plants, conditions of a vegetation room, Figure 7a), nine days (wheat plants, conditions of a vegetation room, Figure 8a), or five days (wheat plants, open-ground conditions, Figure 9a) of the soil drought; the PRI(555,570) decreases were initiated after nine days (pea plants, conditions of a vegetation room, Figure 7b), nine days (wheat plants, conditions of a vegetation room, Figure 8b), or five days (wheat plants, open-ground conditions, Figure 9b) of this drought. It should be additionally noted that the magnitudes of these changes in PRI(515,570) were weak in the investigation of wheat under open-ground conditions (Figure 9a).

**Figure 10:**
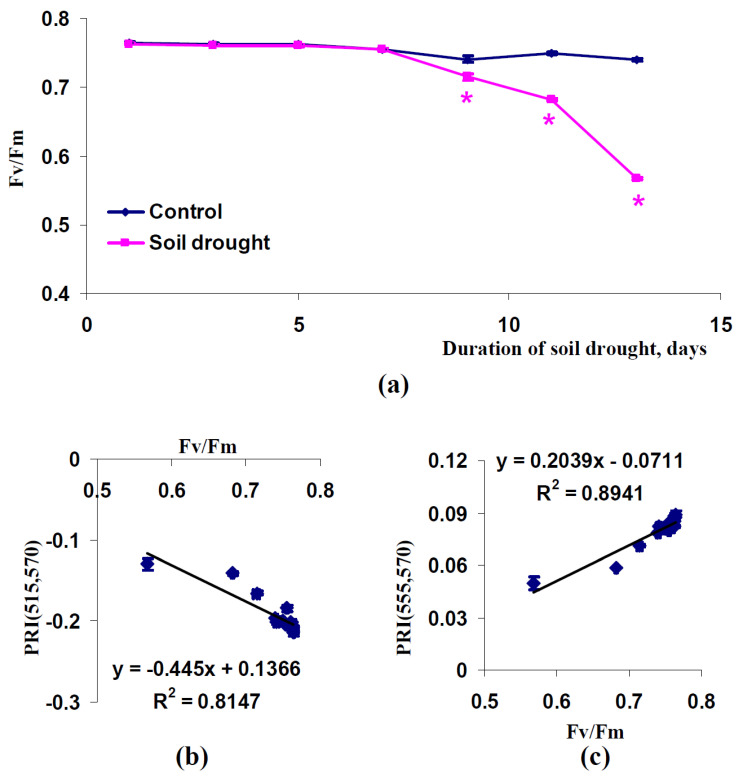
Dependence of Fv/Fm in leaves of pea plants cultivated under conditions of a vegetation room on the duration of the soil drought (*n* = 45) (**a**) and dependences of average values of PRI(515,570) (**b**) and PRI(555,570) (**c**) in the canopy on Fv/Fm (*n* = 14). Average values of PRI(515,570) and PRI(555,570) from Figure 7 were used. R^2^ is the determination coefficient. *, difference between experimental and control indices was significant (*p* < 0.05).

**Figure 11:**
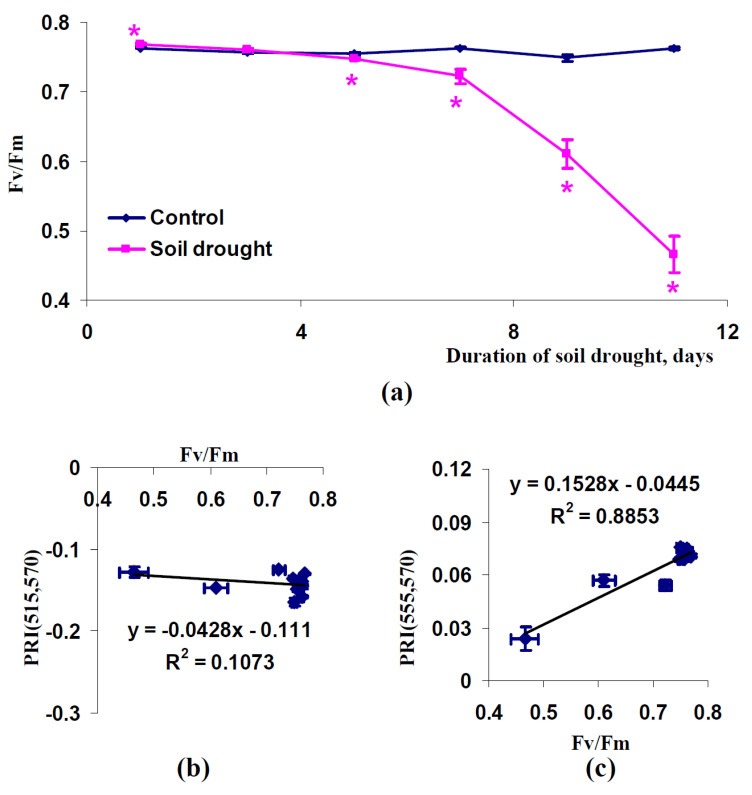
Dependence of Fv/Fm in leaves of wheat plants cultivated under open-ground conditions on duration of soil drought (*n* = 45) (**a**) and dependences of average values of PRI(515,570) (**b**) and PRI(555,570) (**c**) in the canopy on Fv/Fm (*n* = 12). Average values of PRI(515,570) and PRI(555,570) from Figure 9 were used. R^2^ is the determination coefficient. *, difference between experimental and control indices was significant (*p* < 0.05).

It was also shown that soil drought could strongly decrease the maximal quantum yield of photosystem II in pea plants (conditions of a vegetation room, Figure 10a) and wheat plants (open-ground conditions, Figure 11a). Relations between changes in Fv/Fm and PRI(515,570) were varied; a strong relation between these values was observed in pea plants cultivated under conditions of a vegetation room (R^2^ = 0.81) (Figure 10b) and a weak relation between these changes was observed in wheat plants cultivated under open-ground conditions (R^2^ = 0.11) (Figure 11b). Relations of changes in Fv/Fm to PRI(555,570) were strong in all investigated plants; R^2^ equaled 0.88 (pea, conditions of a vegetation room) and 0.89 (wheat, open-ground conditions) (Figure 10c and Figure 11c).

## 3. Discussion

Drought and heat are very important environmental factors influencing photosynthetic processes [11,12,13,14,15]. This influence includes both damage of photosynthetic machinery and induction of photosynthetic-adaptive responses, which can be caused by activation of systems of stress signaling (e.g., signals related to phytohormones, ROS signals, hydraulic signals, electrical signals, and others) [18,19,20,25,26,30,31,33,34]. Development of methods of remote sensing to measure photosynthetic changes caused by action of stressors is an important scientific task contributing to both fundamental and applied photosynthetic investigations. Measurements of spectral characteristics of plant reflectance including multi- and hyperspectral imaging is the perspective method of remote sensing of photosynthetic processes [35,37,42,45,46,69] including their changes under action of stressors.

The photochemical reflectance index is a widely-used reflectance index that can be sensitive to changes in photosynthetic processes under the action of numerous stressors, including intensive light [38,41,43], drought [59,70,71,72], non-optimal temperatures [59,72], nitrogen deficit [73], salt stress [74], etc. Earlier, we showed that the photochemical reflectance index can also be affected by burning-induced electrical signals [60]; the effect is related to the photosynthetic responses induced by these signals. It is important that PRI(531,570) can be sensitive to very fast photosynthetic changes [38,41,43,44,75].

However, the relation between PRI(531,570) and photosynthetic parameters can be strongly varied [42,46,47]. Particularly, values of the photochemical reflectance index can be negatively correlated with NPQ under intensive light and can be positively correlated with one under water deficit [43,44,70]; the last result contradicts the idea about the decrease of PRI(531,570) as the typical response to the action of stressors and the suppression of photosynthesis (including an increase of NPQ and a decrease of Fv/Fm). Different directions of PRI(531,570) changes can be related to their complex mechanisms, including fast transitions in the xanthophyll cycle [38,39,40] and chloroplast shrinkage [41], which are depended on pH in the chloroplast lumen, and long-term changes to the ratio between concentrations of carotenoids and chlorophylls and in the total size of pool of xanthophylls [51,52,53].

Different directions of relations between PRI(531,570) and photosynthetic parameters under the action of different stressors (e.g., intensive light and water deficit) can restrict the use of this index for the remote sensing of photosynthetic stress changes. Potentially, this problem can be minimized by using light-induced changes in the photochemical reflectance index [43,44,59,61,62,63,71,76,77]; however, measurements of these changes have technical limitations. Alternatively, modified photochemical reflectance indices with shifts of the measuring wavelength can be used for solution of this problem. Earlier, we showed that this modification of the photochemical reflectance index can influence its sensitivity to changes in photosynthetic parameters under illumination with high intensity [50]; however, all modified photochemical reflectance indices were decreased under action of intensive light. These results are in good accordance with previous data [38,40].

In the current work, we investigated the influence of water deficit (short-term water shortage and prolonged soil drought) and short-term heat stress on revealed modified photochemical reflectance indices (PRI(515,570), PRI(525,570), PRI(535,570), PRI(545,570), and PRI(555,570)) and on the typical photochemical reflectance index (PRI(531,570)). The main results of the work show that modified photochemical reflectance indices with the longer measuring wavelength (especially PRI(545,570) and PRI(555,570)) were decreased under the action of all investigated stressors (water shortage, soil drought, and heat) in all investigated plants (pea and wheat) at all variants of cultivation (conditions of a growth chamber, conditions of a vegetation room, and open-ground conditions) and at all variants of measurements (spatially-fixed leaves and plant canopy) (Figure 1, Figure 4a, Figure 5a, Figure 6, Figure 7b, Figure 8b and Figure 9b). This decrease is in good accordance with the decreased PRI(545,570) and PRI(555,570) under action of intensive light [50]; moreover, this decrease is in accordance with decreases of the typical PRI(531,570) under various stressors, which were shown in many works [38,39,40,60,74,76,77,78]. Strong relations between PRI(555,570) and the investigated photosynthetic parameters (Figure 4b,c, Figure 10c and Figure 11c) additionally support the efficiency of this index for revealing photosynthetic stress changes. It should be noted that the directions of these relations are in an accordance with the directions of changes in the typical photochemical reflectance index; e.g., both PRI(555,570) in the current work and PRI(531,570) in [41,59,73,74,77,78] are negatively correlated with NPQ.

In accordance with our previous work [50], PRI(545,570) and PRI(555,570) are mainly related to the fast-relaxing component of changes in reflectance, which is predominant in the 535–555 nm spectral range [40,50]; participation of the slow-relaxing component is minimal. Mechanisms of the fast-relaxing component can be related to the light scattering in this spectral range [56,57,58], probably caused by chloroplast shrinkage [41,42]. Considering the dependence of this light scattering [49] and chloroplast shrinkage [41,42] on the luminal pH, changes in this pH can be the key link between changes in PRI(555,570) and photosynthetic parameters, which are strongly related to the pH in the chloroplast lumen [16,26,27,28,54,55,79,80,81]. It is important that the 545–555 nm spectral range is outside of the absorption spectra of the main components of photosynthetic machinery [82] or of the absorption spectra of pigments of the xanthophyll cycle [83]. This means that stressor-induced changes in concentrations of these components and pigments should not influence PRI(545,570) and PRI(555,570); i.e., these changes cannot modify the decrease of PRI(545,570) and PRI(555,570) caused by the decrease of luminal pH and stimulation of light scattering in the green–yellow spectral range under the action of stressors.

By contrast, PRI(515,570) was increased in all variants of our experiments in the current work (Figure 1, Figure 3a, Figure 5a, Figure 6, Figure 7a, Figure 8a and Figure 9a). These results contradict the decrease of PRI(515,570) under illumination with high intensity that was shown in our previous work [50]. Directions of relations of PRI(515,570) to photosynthetic parameters are opposite to directions of the relations for PRI(555,570); the determination coefficients of these relations are lower (Figure 3b,c, Figure 10b and Figure 11b). Moreover, these directions are opposite to the directions of relations between PRI(515,570) and photosynthetic parameters (at least NPQ) under intensive light [50]. This result shows that PRI(515,570) has limited efficiency for the estimation of photosynthetic stress changes because changes to this index and its relations to photosynthetic parameters are dependent on the type of stressor.

Changes in PRI(515,550) are mainly caused by the slow-relaxing component of changes in reflectance, which is predominant in the 515–525 nm spectral range [50]. The mechanism of this component is considered to be related to the de-epoxidation of violaxanthin to zeaxanthin [40,50], which is also dependent on the luminal pH [54]. It can be expected that stressor-induced decrease of the luminal pH should decrease reflectance in the 500–530 nm spectral range [38,40,48]; i.e., PRI(515,550) should be decreased. This effect can be observed under fast photosynthetic changes (minutes) induced by increases of the light intensity [50]. However, the 515 nm wavelength is included in the spectral region of light absorption by the main components of photosynthetic machinery (the cores of photosystems I and II, light-harvesting complex II, and the light-harvesting complex of photosystem I) [82] and pigments of the xanthophyll cycle [83]. It can be hypothesized that the concentration of these complexes and/or these pigments can be decreased for 1 h after short-term heat or at water deficit (short-term water shortage and prolonged soil drought). These decreases should stimulate reflectance at 515 nm and, thereby, increase PRI(515,550); i.e., total changes in this index should be the result of two opposite processes.

Experimental analysis of this hypothesis is outside of the task of the current work because both the high efficiency of PRI(555,570), which is decreased under water shortage, soil drought, and heat stress in the all experimental variants (results of the current work) and under intensive light [50], and the low efficiency of PRI(515,570) are important results for the development of methods of revealing photosynthetic stress changes regardless of the specific mechanisms of changes in these indices.

However, this hypothesis can also explain contradictory results of changes in the typical PRI(531,570). This index is weakly and non-significantly increased by the action of water shortage (Figure 1) and heat (Figure 5a) and is significantly decreased by the action of soil drought (Figure 6). Moreover, the magnitude of decrease of PRI(531,570) in wheat plants (Figure 6b,c) is higher than this magnitude in pea plants (Figure 6a). Based on the hypothesis, it can be proposed that changes in PRI(531,570) are the result of the combination of two processes: a decrease of the typical photochemical reflectance index, which is caused by transition of the xanthophyll cycle and stimulation of light scattering, and an increase of this index, which is caused by a decrease of concentrations of the components of the photosynthetic machinery and the total content of pigments of the xanthophyll cycle. In this case, resulting changes in PRI(531,570) can be strongly affected by moderate changes in experimental conditions. This explanation is in good accordance with results showing participation of transitions in the xanthophyll cycle [35,38,40,45,46,47], modifications of light scattering [49,50], and shifts in concentrations of carotenoids and chlorophylls [51,52,53] in changes in PRI(531,570). Additionally, this hypothesis can be supported by different changes in PRI(531,570) in similar experiments; e.g., we did not show significant changes in this index under the action of water shortage on pea plants in work [50], but showed a significant increase of PRI(531,570) in similar experiments (action of water shortage on pea plants) in work [44].

**Figure 12:**
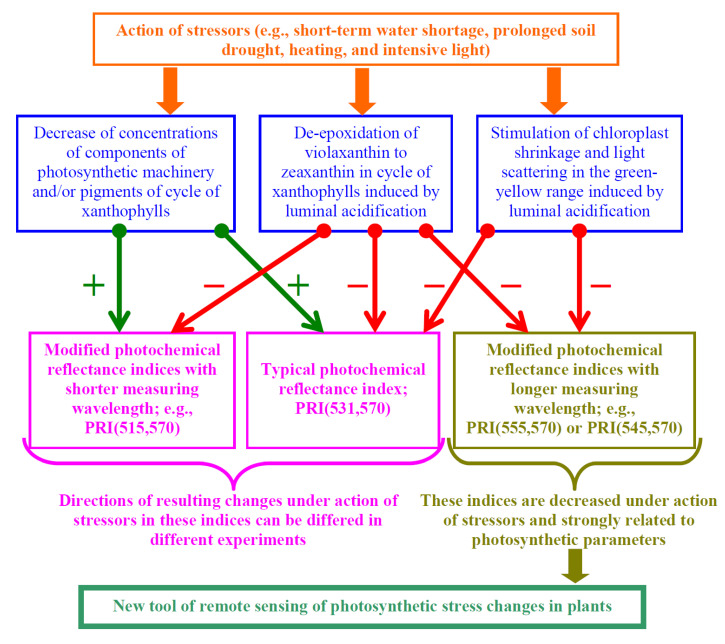
Hypothetical scheme of ways in which stressors influence the typical and modified photochemical reflectance indices. Red and green arrows show negative and positive influence of stressors on these indices, respectively.

Thus, our results show that modified photochemical reflectance indices with the longer measuring wavelength (e.g., PRI(555,570)) are more effective for revealing photosynthetic changes under the action of stressors (short-term heat, water shortage, and prolonged soil drought in accordance with the current work and intensive light in accordance with our previous work [50]) than the typical photochemical reflectance index or modified photochemical reflectance indices with the shorter measuring wavelength (Figure 12). Potentially, these results provide a new tool (PRI(555,570)) for remote sensing of photosynthetic stress changes in plants, including changes induced by the activation of systems of signaling. Other modified photochemical reflectance indices with the longer measuring wavelength (e.g., PRI(545,570), which were decreased in all variants of experiments (Figure 1, Figure 5a and Figure 6) and strongly related to the investigated photosynthetic parameters (data not shown)), can also be this tool.

Finally, perspectives of using this new tool for plant remote sensing based of measurements of modified photochemical reflectance indices should be discussed. First, sensitivity of PRI(555,570) and PRI(545,570) to the action of stressors was observed in both investigated model plants (wheat and pea). Considering the wide-spread use of the typical photochemical reflectance index in the remote sensing of many plant species (e.g., see reviews [42,46,47]), it can be expected that these modified photochemical reflectance indices should be also effective for other plant species. Stressor-induced lumen acidification, which is the basis of changes in PRI(555,570) and PRI(545,570) under the action of stressors [50], seems to be the universal mechanism of the photosynthetic stress response. The last point additionally supports the potential efficiency of PRI(555,570) and PRI(545,570) in plants of different species; however, further experimental investigation of this problem seems to be important.

Second, our results show strong relations of the modified photochemical reflectance indices (PRI(555,570) and PRI(545,570)) to photosynthetic parameters. This means that these indices can be used for the fast estimation of photosynthetic processes, which is similar to using the typical photochemical reflectance index [42,46,47]. Potentially, other important parameters of plants (e.g., growth and yield) can be related to PRI(555,570) and PRI(545,570) (maybe through the relation between photosynthesis and productivity). However, we did not investigate these parameters and their influence on modified photochemical reflectance indices; thus, this question requires future analyses.

Third, it can be expected that measurements of the PRI(555,570) or PRI(545,570) indices in fields can be similar to the measurements of the typical photochemical reflectance index, which are often based on unmanned aircraft systems equipped with multispectral or hyperspectral cameras, or on using handheld devices [35]. From a technical point of view, the transition from PRI(531,570) measurements to PRI(555,570) or, especially, PRI(545,570) measurements seems to be simple (multispectral cameras should be equipped with another narrowband optical filter, and handheld devices should include other LEDs for the measuring light, whereas hyperspectral cameras can be used without additional adaptation); however, development of this equipment is a future task.

Fourth, there are other directions of investigations including the analysis of efficiency of PRI(555,570) or PRI(545,570) for revealing photosynthetic stress changes under action of other stressors and the analysis of characteristics of the spatial distributions of PRI(555,570) or PRI(545,570), which will be directed to increasing the accuracy of remote sensing of photosynthetic parameters and revealing markers of actions of specific stressors.

## 4. Materials and Methods

### 4.1. Analysis of Influence of Short-Term Water Shortage and Heat Stress on Modified Photochemical Reflectance Indices in Spatially-Fixed Leaves of Pea Plants

We used the spectra of reflected light and photosynthetic parameters, which were measured in experiments for our previous work [59]; only unpublished data were used.

Two-to-three-week-old pea plants (*Pisum sativum* L., cultivar “Albumen”) were used for this investigation. Plants were cultivated in a Binder KBW 240 plant growth chamber (BinderGmbH, Tuttlingen, Germany) at 24 °C under a 16/8 h (light/dark) photoperiod. A sand substrate with irrigation (every two days) was used for the plant cultivation in the investigation of short-term water shortage’s influence, and 50% hydroponic Hoagland–Arnon medium based on chemicals from Sigma-Aldrich (St. Louis, MO, USA) was used for plant cultivation in the investigation of the influence of short-term heat. Water shortage was induced by termination of irrigation; this termination decreased water content in plants and induced withering for five days. In experiments with short-term heat, pea plants were heated in the thermostat TV-20-PZ-“K” (Kasimov Instrument Plant, Kasimov, Russia) at 46.5 °C for 30 min. Photosynthetic and reflectance measurements were performed every day in experiments with water shortage or 1 h after termination of action of the increased temperature in experiments with heat stress. Five to six plants were investigated in each experimental point.

The PAM-fluorometer Dual-PAM-100 (Heinz Walz GmbH, Effeltrich, Germany) was used for photosynthetic measurements. Fv/Fm was measured after 15 min dark adaptation; NPQ was measured after 8 min of illumination by white actinic light (the halogen lamp, Osram Decostar, 3000 K, 20 W, 12 V, Germany) with 630 µmol m^−2^ s^−1^ intensity. Intensities of reflected light were measured simultaneously with the NPQ measurement after 8 min of illumination by actinic light.

The S100 spectrometer (SOLAR Laser Systems, Minsk, Belarus), connected with a fiber-optic cable, was used for measurements of spectra of reflected light; a 18% grey card (QPcard 101 Calibration Card v3, Argraph Corp., Carlstadt, NJ, USA) was used as the reflectance standard. Modified and typical photochemical reflectance indices (PRI(λ,570)) were calculated on the basis of Equation (2) in accordance with [50]:

(2) $$\mathrm{PRI}\left(\lambda ,570)\right)=\frac{R_{\lambda }-R_{570}}{R_{\lambda }+R_{570}},$$

where R_λ_ and R_570_ are reflectance at λ nm (the measuring wavelength) and 570 nm (the reference wavelength), respectively. λ was 515, 525, 535, 545, and 555 nm for modified photochemical reflectance indices and was 531 nm for the typical photochemical reflectance index.

### 4.2. Analysis of Influence of Prolonged Soil Drought on Modified Photochemical Reflectance Indices in Canopy of Pea and Wheat Plants

We used the spectra of reflected light and photosynthetic parameters, which were measured in experiments for our previous work [65]; only unpublished data were used.

Two-to-four-week-old pea (*Pisum sativum* L., variety “Albumen”) and wheat (*Triticum aestivum* L., variety “Zlata”) plants were used in experiments. There were two variants of plant cultivation. (i) Pea and wheat plants were cultivated in a vegetation room (a 16/8 h (light/dark) photoperiod, 24 °C). (ii) Wheat plants were cultivated under open-ground conditions (Nizhny Novgorod, July 2021, 18 h light day, 27 °C (averaged day temperature) and 18 °C (averaged night temperature)). Plants were cultivated in pots (nine plants per pot); pots were placed on pallets to imitate canopy for analysis. Forty-five pots were investigated in each experimental point. The soil drought was initiated by termination of irrigation and continued until withering; control plants were irrigated (every two days). Natural irrigation under the open-ground conditions was excluded.

Hyperspectral images of the canopy were obtained in control and experimental plants every two days after the soil drought initiation. A Specim IQ hyperspectral camera (Specim, Spectral Imaging Ltd., Oulu, Finland) was used for measurements; a white reference panel was used as the reflectance standard. Reflectance of plants was measured at a 45° angle to the ground plane. Illumination of luminescent lamps FSL YZ18RR (Foshan Electrical And Lighting Co., Ltd., Foshan, China) and sunlight was used as the incident light in measurements of reflectance spectra of plants cultivated in the vegetation room and under open-ground conditions, respectively.

We used programs that were specially developed for this task using the Python coding language (libraries Spectral and Numpy), for excluding the background on the basis of reflectance at 543 nm (the high leaf reflectance and low light absorption) and 661 nm (the low leaf reflectance and high light absorption by chlorophylls) and for the calculation of modified and typical PRI(λ,570) in accordance with Equation (2).

Maximal quantum yields of photosystem II were measured using the handheld PAM fluorometer PAR-FluorPen FP110 (Photon Systems Instruments, Drasov, Czech Republic) under conditions with low intensity of light. Fv/Fm were measured in three leaves of different plants in each pot; after that, these values were averaged. Forty-five pots were investigated in each experimental point.

### 4.3. Statistics

Mean values, standard errors (SEs), scatter plots, linear regression equations, and determination coefficients are shown in the figures. Numbers of repetitions are shown in the figures. Significance of differences was estimated using Student’s *t*-test (Microsoft Excel 365).

## 5. Conclusions

Soil drought and heat are important stressors suppressing photosynthetic processes in plants and regulating these processes through induction of adaptive responses (maybe with the activation of systems of stress signaling). The photochemical reflectance index, which is related to photosynthetic processes, is considered to be an effective tool for revealing the action of stressors on plants; however, this efficiency can be strongly dependent on specific experimental conditions. In the current work, we analyzed changes in modified photochemical reflectance indices under water deficit and heat stress.

It was shown that the indices with the longer measuring wavelength (e.g., PRI(555,570)) are more effective for revealing the action of stressor than the typical photochemical reflectance index and modified photochemical reflectance indices with shorter measuring wavelength, because these indices were decreased under action of water shortage, soil drought, heat and intensive light at all variants of experiments. This result provides a new tool (PRI(555,570) and similar modified photochemical reflectance indices, e.g., PRI(545,570)) for remote sensing of photosynthetic stress changes in plants, including changes induced by activation of systems of signaling.

## Data Availability

The data presented in this study are available upon request from the corresponding author.
